# Preconception Screening for Cytomegalovirus: An Effective Preventive Approach

**DOI:** 10.1155/2014/135416

**Published:** 2014-06-12

**Authors:** Orna Reichman, Ian Miskin, Limor Sharoni, Talia Eldar-Geva, Doron Goldberg, Avi Tsafrir, Michael Gal

**Affiliations:** ^1^Department of Obstetrics & Gynecology, Shaare Zedek Medical Center, Hebrew University Medical School, Jerusalem, Israel; ^2^Clalit Health Services, Jerusalem, Israel; ^3^Department of Clinical Microbiology and Infectious Diseases, Hadassah-Hebrew University Medical Center, Ein Kerem, Jerusalem, Israel; ^4^Departments of Anesthesiology and Critical Care, Hadassah-Hebrew University Medical Center, Ein Kerem, Jerusalem, Israel

## Abstract

Congenital cytomegalovirus (CMV) is the leading infectious cause of sensorineural hearing loss and delayed psychomotor development. Viral transmission to the fetus is far more likely to occur following a primary than a secondary maternal infection. Primary prevention seems to be the best means to reduce the burden of congenital CMV due to the lack of treatment options during pregnancy. We evaluated this approach on a cohort of 500 women planning pregnancy who attended our fertility clinic. Of the 444 who underwent CMV screening, 18 (4.1%) had positive IgM serology for CMV; of these, IgG avidity was high in 12 (remote infection) and low in 6 (recent infection). The latter were advised to delay pregnancy. All women who were seroimmune for CMV (366/444, 82.4%), including the 12 with remote infection, continued fertility treatment. The remaining patients (72/444, 16.2%), who were not immune to CMV at the initial screen, were advised to minimize CMV exposure by improving personal hygiene and to continue fertility treatment. None of the 69/72 (95.8%) women who were followed for one year were infected with CMV. Cytomegalovirus testing and counselling at preconception seemed effective in reducing CMV exposure in pregnancy.

## 1. Introduction

Congenital cytomegalovirus (CMV) is the leading infectious cause of sensorineural hearing loss and delayed psychomotor development. The annual health care burden exceeds 1.86 billion dollars in the US [1–5]. The birth prevalence of congenital CMV varies considerably among populations and ranges between 0.2 and 2% [6, 7].

Congenital CMV results from hematogenous dissemination following maternal viremia. Viral transmission to the fetus is far more likely to occur following a primary maternal infection than a secondary infection (relative risk of 30–40) [6, 8]. Secondary infection results from an infection with a new strain of CMV or reactivation of a previously infecting strain. In both cases the presence of humeral and cell-mediated immune elements reduces the likelihood of fetal infection from 30–40% to 1–5% [9]. Timing of maternal CMV infection is crucial in predicting clinical outcome. Although viral transmission is higher with advanced stages of pregnancy, clinical sequels are poorer for fetuses infected in early stages of pregnancy [10–13].

Two antepartum therapeutic options for treating a fetus infected with CMV are currently available: intravenous maternal CMV-specific hyperimmune globulin and direct administration of ganciclovir to the umbilical vein; however, there is limited experience with these interventions and the outcomes have not demonstrated efficacy in randomized controlled studies [2, 14–16]. Due to the potential hazards of primary maternal infection in early pregnancy and the limited treatment options, primary prevention appears to be the best means to reduce the burden of congenital CMV. These include vaccination [4, 17], preconception screening [18, 19], and reinforcing behavior that should reduce exposure to viral shedding, such as thorough hand-washing after exposure to children at home and in day-care centers [5, 20].

Maternal preconception screening for CMV can identify women with three categories of CMV serotypes, all of whom will benefit from specific counseling: first, women with recent seroconversion will benefit by postponing their pregnancy; second, those with remote infection or reactivation will be able to continue fertility treatment without the need for further invasive procedures such as amniocentesis; and third, seronegative women who are at risk for seroconversion during pregnancy will learn how to reduce their exposure to viral shedding by adopting simple measures [20]. Despite routine screening for CMV as part of the preconception visit [20–22], solid evidence for this approach is lacking.

In our fertility clinic we screen all women for various infectious diseases including CMV, as part of preconception counseling. The objective of this study is to describe the results of this initial screening for CMV, the effect of the resulting counseling, and the outcome of follow-up of the seronegative patients.

## 2. Materials and Methods

We performed a retrospective cohort study of 500 consecutive women who were referred to our outpatient fertility clinic at Shaare Zedek Medical Center, Jerusalem, during a 28-month period. At the initial visit, as part of the preconception counselling of a planned pregnancy, demographic data, occupation, past medical history, and assessment of the patient's risk for acquiring CMV (working in a day-care center and/or having children of ages 1–4 years at home) were recorded. All women were referred for preconception blood test screening for CMV by LIAISON ELISA kits (LIAISON CMV-IgG and -IgM, DiaSorin, Italy). At the 2–4-week follow-up visit, immunization status was classified as one of the following: positive: past history of CMV infection, (IgG(+)/IgM(−)); negative: no history of exposure to CMV (IgG(−)/IgM(−)); and intermediate: further divided into current primary infection (IgG(+) low avidity/IgM(+)) and past primary/reactivation of CMV infection (IgG(+) high avidity/IgM(+)). For the patients in the intermediate category, additional consultation was given by the infectious disease specialist (IM), mainly as to whether to delay planned pregnancy or to use contraception in the meantime. For seronegative women, as a rule, preconception counselling included explanatory measures to improve personal hygiene, especially hand-washing after exposure to infants and young children at home or in day-care centers [5, 20], and follow-up by further CMV serology tests every 3-4 months for at least a further year, during infertility treatment and pregnancy (if achieved).

The study protocol was approved by the Shaare Zedek Medical Center IRB (Helsinki) Committee.

## 3. Results

Five hundred women who planned to conceive and who applied to our clinic for infertility investigation were referred, on their first visit at the clinic, for CMV evaluation as part of preconception counselling. Of these, 444 complied with the recommendations and underwent blood tests to evaluate their CMV immunity status. The other 56 patients deferred their blood test and/or did not return to the fertility clinic.

Table 1 summarizes the demographic characteristic of the population. The majority of women (72.3%) were at low risk for acquiring CMV infection.

**Figure 1:**
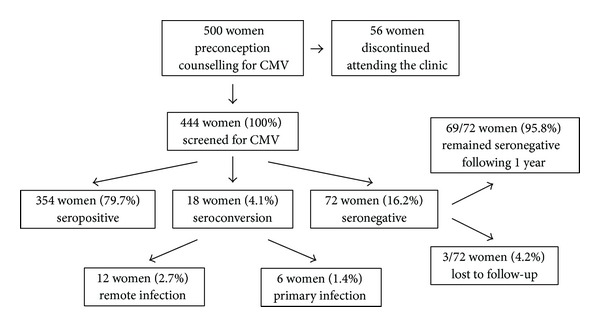
Immune status for CMV at preconception screening.

Figure 1 presents the screening results of CMV serology for the study population. Most women (79.7%) were seropositive (IgG(+)/IgM(−)) and were not further evaluated for CMV serology. The 72 women (16.2%) who were seronegative (IgG(−)/IgM(−)) were advised to adopt behaviors that would reduce exposure to viral shedding, such as thorough hand-washing after exposure to young children at home and/or in day-care centers. In addition, they were advised to undergo follow-up evaluation of their CMV immunity status every 3-4 months. Of the seronegative patients, 69 underwent further serology testing for CMV; no patient showed seroconversion during the one year following the start of screening. The other 3 patients were lost to follow-up.

At baseline screening, 18 women (4.1%) were found to have evidence of seroconversion. Of them, 12 were categorized as past primary CMV infections or reactivations (IgG(+) high avidity/IgM(+)); they continued with infertility treatment. Six women (1.4%) were diagnosed with primary infection (IgG(+) low avidity/IgM(+)) and were advised to postpone pregnancy for 6–9 months. All six complied with the recommendations of the infectious diseases consultant and postponed their infertility treatment and commenced active contraception.

## 4. Discussion

In this historical prospective study, we evaluated a cohort of 500 patients who planned pregnancy and were referred to a fertility clinic. Preconception screening for CMV identified 6/444 women (1.4%) with recent primary infection (seroconversion). Postponing infertility treatment prevented exposure to primary CMV infection in early pregnancy, with all the associated consequences: maternal anxiety regarding fetal sequels, need for amniocentesis (to rule out fetal viral transmission), and sequential ultrasound every 3-4 weeks with additional MRI directed to identify the effects of intrauterine CMV [2]. Most importantly, postponing pregnancy averted the complications of primary CMV infection in early pregnancy in 1.4% of the study population.

The sequels of congenital CMV infection can be severe. Of infants who are congenitally infected in the first half of pregnancy 10–15% are born with symptoms: intrauterine growth retardation, microcephaly, jaundice, petechiae, chorioretinitis, thrombocytopenia, and/or hepatosplenomegaly, with a mortality rate of 20–30% [3]. Of the 85–90% who are asymptomatic at birth, 10–15% develop hearing loss and delayed psychomotor development [3, 13]. To estimate the burden of congenital CMV infection in a population, one needs to know the prevalence of congenital CMV and the timing of infection; the latter is crucial to predicting clinical outcome. A population prevalence rate of congenital CMV of 1% was previously reported for our medical center [23]. Assuming that 50% of the congenital CMV infants would be infected in the first trimester, of whom 90% would be asymptomatic, and 10% would develop sequels later in life, and 10% would be born with symptoms, of whom 90% would suffer from sequels, then an estimated 9 of 10,000 infants who are born in our medical center will suffer from a sequel of congenital CMV infection. Our obstetric division is one of the largest in the country, with 20,000 deliveries a year; thus about 18 infants born at our center annually would be expected to develop sequels of congenital CMV. This calculation is in accordance with a meta-analysis of 27 studies that estimated a rate of symptomatic congenital CMV as 7 of 10,000 live births [6].

The detection of CMV seronegativity in 16.2% of women in the current study is congruent with a separate report of 20% seronegativity at our medical center [23]. Other publications have reported conversion during pregnancy in 1–4% of seronegative women, as a result of primary CMV infection [8]. Thus, it is estimated that preconception screening of 500 women in our population will detect 2 women with primary infection. We screened 448 women and detected primary CMV seroconversion in 6. The similarity between the expected and the actual numbers strengthens the internal validity of our study.

This study only followed women for whom screening showed evidence of CMV seronegativity or seroconversion; seropositive women were not followed for possible reinfection or reactivation. Thus, compatible with the policy of the Israel Ministry of Health and the CDC, assessment of the burden of secondary infection was not within the scope of this study. Moreover, the high compliance rate, 95.8% of seronegative women who repeated CMV testing every 3-4 months, may be consequent to the infertility treatments that require repeated hormonal blood tests and visits at the fertility clinic. Studies are thus necessary to assess if advice on improving adherence to personal hygiene is as effective in preventing primary infection with CMV in the general population of reproductive women as it is among women receiving fertility treatment.

## 5. Conclusions

Preconception screening for CMV has the potential to advise postponing pregnancy for women with recent infection and to guide in preventive measures for those who are seronegative. As an adjunct to planned pregnancy, preconception counseling enables minimization of the effects of infectious diseases.

## Figures and Tables

**Table 1:** Characteristics of 444 women who performed preconception screening for CMV.

Mean age, years	29.9 ± 3.4
Mean number of children	0.7 ± 1.1
Place of Birth, *n* (%)	
Israel	397 (89.4)
Outside of Israel	47 (10.6)
Religion, *n* (%)	
Orthodox Jews	234 (52.7)
Secular Jews	96 (21.6)
Arabs (Muslim, Christian)	31 (7.0)
Unknown	83 (18.7)
Risk for CMV infection, *n* (%)	
^a^High risk	98 (22.1)
^b^Low risk	321 (72.3)
Unknown	25 (5.6)
Residence, *n* (%)	
Urban	420 (94.6)
Rural	24 (5.4)
Female infertility, *n* (%)	
Primary	233 (52.5)
Secondary	211 (47.5)

^a^High risk: work in a day-care center or with children aged 1–4 years old at home.

^b^Low risk: all others.
